# *N*-Acetylcysteine an *Allium* Plant Compound Improves High-Sucrose Diet-Induced Obesity and Related Effects

**DOI:** 10.1093/ecam/nen070

**Published:** 2011-02-20

**Authors:** Gisele A. Souza, Geovana X. Ebaid, Fábio R. F. Seiva, Katiucha H. R. Rocha, Cristiano Machado Galhardi, Fernanda Mani, Ethel L. B. Novelli

**Affiliations:** ^1^Department of Chemistry and Biochemistry, Institute of Biosciences, São Paulo State University, UNESP, 18618-000, Botucatu, São Paulo, Brazil; ^2^Post Graduation Course, Department of Clinical and Cardiology, School of Medicine, São Paulo State University, UNESP, Botucatu, São Paulo, Brazil

## Abstract

This study was designed to determine whether *N*-acetylcysteine (NAC, C_5_H_9_–NO_3_S), a compound from *Allium* species may be used as a complementary therapeutic agent, to inhibit high-sucrose induced-obesity and its effects on glucose tolerance, *in vivo* low-density lipoprotein (LDL)-oxidation and serum oxidative stress in rats. Initially, 24 male Wistar rats were divided into two groups: controls receiving standard chow (C, *n* = 6) and those receiving high-sucrose diet (HS, *n* = 18). After 22 days, (HS) group was divided into three groups (*n* = 6/group); (HS-HS) continued to eat high-sucrose diet and water; (HS-N) continued to eat high-sucrose diet and received 2 mg l^−1^-NAC in its drinking water; (HS-CN) changing high-sucrose to standard chow and receiving 2 mg l^**­**1^-NAC in its drinking water. After 22 days of the HS-group division (44 days of experimental period) body weight, body mass index and surface area were enhanced in HS-HS rats (*P* < .001). HS-HS rats had glucose intolerance, increased serum triacylglycerol (TG), very low-density lipoprotein (VLDL), oxidized-LDL (ox-LDL) and lipid-hydroperoxide (LH) than the others (*P* < .01). NAC in HS-N and HS-CN rats reduced the obesity markers, feed efficiency, LH and ox-LDL, as well normalized glucose response, TG and VLDL (*P* < .01) in these groups compared with HS-HS. Total antioxidant substances, GSH/GSSG ratio and glutathione-reductase, were higher in HS-N than in HS-HS (*P* < .01). In conclusion, NAC improved high-sucrose diet-induced obesity and its effects on glucose tolerance, lipid profile, *in vivo* LDL-oxidation and serum oxidative stress, enhancing antioxidant defences. The application of this agent may be feasible and beneficial for high-sucrose diet-induced obesity, which certainly would bring new insights on obesity-related adverse effects control.

## 1. Introduction

The use of plant compounds in managing of pathology has increased in recent years, as adjunct therapy and alternative medicine. In view of the fact that reactive oxygen substances (ROS) are implicated in the etiology of many degenerative diseases; antioxidants have become one among the most important topics in nutrition.

*N*-Acetylcysteine (NAC, C_5_H_9_–NO_3_S), an organosulfur from *Allium* plant, due to its potential antioxidant activity has been considered responsible, at least in part, for onion and garlic beneficial effects in warding off illnesses and cardiovascular protection [1, 2]. It is a thiol agent whose biological importance has crossed the boundaries of botany, pharmacognosy and natural products [3, 4]. NAC has been successfully used as adjunct therapy in various pulmonary disorders [5], to improve hepatic function [6], to reduce metal toxicity [7], but the mechanisms responsible for these activities are not yet completely understood.

Recently, we have reported the preventive effects of NAC on dyslipidemic profile and alleviation of hyperglycemia in rats given standard chow and 30% sucrose in its drinking water [8]. It was also demonstrated NAC beneficial effects on sucrose-induced insulin resistance and oxidative stress [9, 10]. However, its effect on obesity-related metabolic changes due high-sucrose intake, as a food compound is not presently known. An increasingly preference for refined foods and diet containing high levels of sucrose has resulted in a drastic increase in the incidence of obesity. Dietary sucrose is an important modulator of the obesity-related diseases [11], but whether dietary NAC can reverse these adverse effects merits to be investigated.

It is well accepted that obesity exerts deleterious effects on health due the remarkable abilities to induce oxidative stress, an imbalance between oxidants and antioxidants systems in favor of the former. During energy metabolism, the mitochondrial respiratory chain represents a major intracellular source of ROS. As the use of oxygen is vital for oxidative phosphorylation, alterations in food constituents or fuel for energy generation may result in higher ROS production, thus inducing oxidative stress [12].

Moreover, oxidative modification of low-density lipoprotein (LDL) producing oxidized-LDL (ox-LDL) is the key step in the sequence of events leading to atherosclerosis. Researches on NAC actions have been reported in a scattered fashion [9, 13–15], but its effects on lipid profile and *in vivo* ox-LDL had drawn little attention until recently. Since the majority of metabolic changes related to oxidative stress and obesity, remain clinically silent and only become manifest when health damage is effectively installed, studies to discover the relative potency of NAC on LDL-oxidation *in vivo* may have particular importance.

Thus, the major purpose of the present study was to examine whether dietary *N*-acetylcysteine (NAC) supplementation may improve high-sucrose diet-induced obesity and its effects on glucose tolerance, lipid profile, *in vivo* LDL-oxidation and serum oxidative stress.

## 2. Methods

### 2.1. Animals

All experiments and procedures were performed in accordance with the Guide for the Care and Use of Laboratory Animals published by the US National Institute of Health, and approved by the Ethics Committee of The Institute of Biosciences, São Paulo State University, UNESP, Brazil. Male Wistar 24 rats, 60 days of age, weighing 202.8 ± 5.0 g were individually housed in polypropylene cages in an environmentally controlled, clean-air room with a temperature of 22 ± 3°C, 12 h light:dark cycle, and a relative humidity of 60 ± 5%.

### 2.2. Obesity Induction and Experimental Design

Initially the animals were randomly divided into two groups. A control (C, *n* = 6) group received standard rodent chow (BioBase, São Paulo, Brazil), containing 23.54% protein, 43.88% carbohydrate (40.75% starch and 3.13% sucrose), 1.87% fat, 13.85% fiber (by weight) and 2.86 kcal g^−1^ of metabolizable energy. The (HS, *n* = 18) group received high-sucrose diet containing (by weight) 23.60% protein, 49.85% carbohydrate (24.5% starch and 27.35% sucrose), 3.27% fat, 9.39% fiber and 3.23 kcal g^−1^ of metabolizable energy. Food intake and drinking solutions consumption were evaluated daily at the same time (9:00-10:00 h), as the difference between food and drinking solution given and the leftover. The body weights were determined once a week.

The high-sucrose diet was obtained mixing 600 g sucrose and 60 g of soy oil to 1000 g of a previously triturated standard chow. Casein was added to achieve the same protein content as the standard chow. The dietary ingredients were homogenized in 60°C warm distilled water and the homogenate was used to prepare the pellets. Therefore, both control and experimental diets were given fresh each day as dry pellets, and there was no spillage. The proportions of dietary protein, carbohydrate and fat were determined by direct analysis [16].

After 22 days of dietary treatments, the animals were anaesthetized (0.1 ml ip of 1% sodium barbiturate) for the measurement of body length (nose-to-anus, or nose-anal length). The body weight and body length were used to confirm the obesity through the obesity parameters, body mass index (BMI, g/cm^2^) = body weight/length^2^, surface are (g^0.7^) = body weight^0.7^ and the Lee-index (g cm^*­*1^) = cube root of body weight/length [17].

In order to more appropriately study the effects of NAC on high-sucrose diet-induced obesity, the (HS) group was then randomly divided into three subgroups (*n* = 6/group): (HS-HS) group, continued to eat high-sucrose diet and received water; (HS-N) group continued to eat high-sucrose diet and received NAC (2 mg l^−1^) in its drinking water; (HS-CN) group that changed high-sucrose diet to standard-chow and received 2 mg l^−1^ NAC in its drinking water. Rats in the C group (*n* = 6) remained with standard chow and water during all experimental period. The HS-CN group was adopted to evidence whether there is synergistic effects between NAC intake and changing high-sucrose diet to control chow, on obesity adverse effects. The dose of NAC used was equivalent to 32.2–39.2 mg/day in humans (70 kg) and was calculated from NAC concentration in onion (*Allium cepa*, 45 mg kg^−1^) [2]. This dose corresponded to intake one onion/day in humans [8]. The food intake and body weight were used to determine the energy intake (EI, kcal/day) = (mean food consumption per day × dietary metabolizable energy) and feed efficiency (FE%) = (mean body weight gain × 100/energy intake).

### 2.3. Oral Glucose Tolerance Test and Glycemic Response

After 22 days of the (HS) group division (44 days of the experimental period) all animals were fasted for 12–14 h and submitted to the oral glucose tolerance test (OGTT). So, glucose was orally given (gastric tube) (2 g kg^−1^), as a 20% aqueous solution. Blood glucose levels were measured before the glucose administration and at 30, 60, 90, 120 and 150 min after the glucose loading. Blood was taken from a tail vein and glucose concentrations were determined by an automatic glucose analyser (Boehringer Mannheim, Eli Lilly Ltda, São Paulo, Brazil). The glycemic response was calculated from the area under the glucose-response curve above the fasting level [11], as a mean to estimate release insulin in response to glucose [18]. After the OGTT, the animals were anaesthetized (0.1 ml ip of 1% sodium barbiturate) for measurement of the body length [17], and were sacrificed by decapitation.

### 2.4. Serum Determinations

Total blood was collected by a funnel into a centrifuge tube and allowed to clot to obtain the serum. Serum was separated by centrifugation at 1400 g for 10 min. Triacylglycerol (TG), total cholesterol and high-density lipoprotein cholesterol (HDL) were determined in serum by enzymatic method (test Kit CELM, Modern Laboratory Equipment Company, São Paulo, Brazil). Total antioxidant substances (TAS), or total antioxidant capacity of serum (Test Kit Randox Laboratories Ltd, Crumlin, Co. Antrim, UK), total protein [19], LDL [20], ox-LDL [20, 21] and lipid-hydroperoxide (LH) [21] were also determined in serum.

The glutathione antioxidant system was assessed in serum by glutathione reductase (GSH-reductase, E.C.1.6.4.2) activity, oxidized glutathione (GSSG) and reduced glutathione (GSH). GSH-reductase activity was evaluated monitoring NADPH (reduced nicotinamide adenine dinucleotide phosphate) oxidation at 340 nm [22]. The assay mixture contained 1 mM Tris buffer, pH 8.0, 5 mM EDTA, 33 mM GSSG and 2 mM NADPH. GSSG was assayed with 2 mM 5,5′-dithiobis-(2-nitrobenzoic) acid (DTNB) in 50 mM KH_2_PO_4_. GSH was measured by a kinetic assay in reaction medium containing 0.6 mM DTNB, 0.2 mM NADPH and 2 U of glutathione reductase in 50 mM KH_2_PO_4_ [23].

Enzyme activities were performed at 25°C using a micro-plate reader (*μ*Quant-MQX 200, Kcjunior software, Bio-Tec Instruments, Winooski, VT, USA). The spectrophotometric determinations were performed in a Pharmacia Biotech spectrophotometer with temperature-controlled cuvette chamber (UV/visible Ultrospec 5000 with Swift II applications software to computer system control, 97 4213, Cambridge, England, UK).

### 2.5. Statistical Analysis

Analysis of variance was used to examine the treatment effect and comparison between the means was performed by Tukey's test, with 0.05 as the significant level (SigmaStat software for windows, Jandel Corporation, San Rafael, CA, USA).

## 3. Results

### 3.1. Obesity Markers

After 22 days of the experimental period, the body weight, BMI, Lee-index and surface area were higher in HS rats than in C (*P* < .001) (Table 1).

### 3.2. General Characteristics of Rats, Nutritional Parameters and Glucose Response

Body weight gain and final body weight, feed efficiency, BMI and surface area were higher in HS-HS rats than in the others. Food consumption, energy intake and drinking solution ingested were lower in HS-HS, HS-N and HS-CN groups than in C. HS-HS rats had the highest FE. NAC normalized FE comparing HS-N and HS-HS rats (*P* < .01). FE was reduced in HS-CN rats as compared with HS-HS (*P* < .01), but was enhanced comparing HS-CN and C groups (*P* < .01). There were no significant differences in NAC intake between HS-N and HS-CN rats. The glycemic response was higher in HS-HS rats than in the others (Table 2).

**Figure 1:**
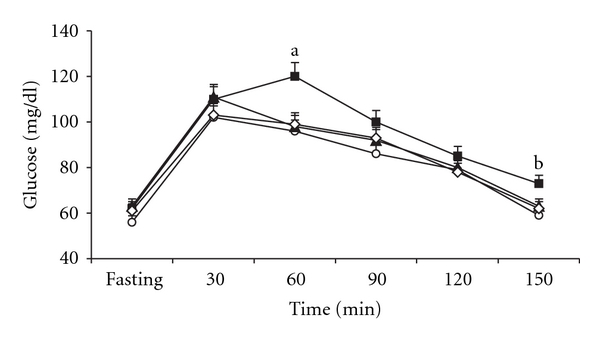
Mean levels of glucose in the oral glucose tolerance test of control rats (C) (open circle), rats receiving high-sucrose diet and water (HS-HS) (filled square), rats receiving high-sucrose diet and water during 22 days, giving NAC in its drinking water for more than 22 days (HS-N) (filled triangle), rats receiving high-sucrose diet and water during 22 days, giving NAC and control chow for more than 22 days (HS-CN) (open diamond). (a) Values significantly different from HS-N, HS-CN and C groups, *P* < .05. (b) Values significantly different from its fasting blood glucose level *P* < .05.

The fasting blood glucose levels were comparable in all four groups. The HS-HS rats had 60-min overload glycemia in relation to the fasting condition. For C, HS-N and HS-CN groups, the glucose level peaked at 30 min, and dropped thereafter. Glucose levels after OGTT were higher than the respective fasting glucose levels in HS-HS rats. The glycemia returned to the fasting levels at 150 min after the glucose administration in HS-N and HS-CN rats (Figure 1).

### 3.3. Lipid Profile and Oxidative Stress in Serum

Triacylglycerol, VLDL, LDL, ox-LDL were significantly higher, while HDL and HDL/TG ratio were lower in HS-HS than in C rats (*P* < .01). NAC normalized TG and VLDL, as well reduced total cholesterol, LDL and ox-LDL, comparing HS-N and HS-HS rats (*P* < .01). HDL/TG ratio was higher in HS-N than in HS-HS rats (*P* < .01). The combination of NAC intake and diet changing from high-sucrose to standard chow in HS-CN had no effects on LDL concentration, but normalized TG, cholesterol, HDL, VLDL and HDL/TG ratio. Ox-LDL was lower (*P* < .01) in HS-CN than in HS-HS, but there were no significant changes in ox-LDL of HS-N and HS-CN rats.

Analysing the oxidative stress markers, we can see that LH, GSH-reductase were higher, while TAS and GSH/GSSG ratio were significantly lower in HS-HS than in C rats (*P* < .01). LH was lower, while TAS, GSH-reductase and GSH/GSSG ratio were higher in HS-N than in HS-HS and C rats. There were no significant differences in LH and GSH-reductase comparing HS-CN with C. TAS and GSH/GSSG ratio were higher in HS-CN than in C and HS-N groups (Table 3).

## 4. Discussion

Taking into account the association of oxidative stress and various pathological conditions, much attention is being paid on dietary antioxidants as alternative therapeutic agents. To the best of our knowledge this is the first study that evaluated the NAC effects on oxidative stress, lipid profile and serum *in vivo* ox-LDL in rats previously submitted to experimental obesity due high-sucrose chow intake. Recently we have reported the beneficial effects of NAC in rats receiving both NAC and 30% sucrose in its drinking water for 30 days. In the present study, NAC was given to obese rats to find out whether NAC intake could reduce the obesity-adverse effects. The rate of obesity in general population has been attributed to sucrose added to dry food, as given in this experimental model.

The increased BMI, surface area, Lee-index and final body weight in HS group (Table 1), demonstrated the efficiency of high-sucrose intake to induce obesity in these animals. Considering food intake of the C and HS-HS groups (Table 2) with a 15% decrease in daily energy intake in the later group, there was an unexpected increased weight gain in HS-HS animals. Curiously, the food consumption and energy intake were significantly similar in the HS-HS, HS-N and HS-CN groups (Table 2), while obesity was only evidenced in HS-HS rats. This observation indicated that dietary factors other than energy intake play important role in body weight regulation. The effect of dietary energy intake on weight gain has been questioned in recent years [24], and energetic-restricted diets have shown poor long-term effectiveness in body weight loss [25].

Previous studies in our laboratory demonstrated that addition of sucrose to drinking water enhanced the energy intake in spite of the lower food consumption [11]. Therefore, a lower energy intake from chow was compensated by additional calories from the aqueous sucrose solutions. Although the two experimental models induced obesity in rats, the discrepancy in the energy intake was attributed to the source of dietary sucrose. Results concerning weight gain in sucrose-fed rats are not entirely conclusive, and high-sucrose diet-induced obesity was found even in absence of increased energy intake [26]. Note that the feed-efficiency was highest in HS-HS rats indicating enhanced capacity for energy conversion and storage. This fact indicated that one mechanism by which the body weights were increased in spite of reduced energy intake in HS-HS group compared with C was the higher feed efficiency. NAC reduced the feed-efficiency and the body weight gain (Table 2).

It was previously observed that high-sucrose diet-induced obesity and impaired the glucose response [8]. Note that HS-HS rats had high-glycemic response (Table 2) and high-blood glucose after the OGTT, indicating decreased rate of glucose utilization and that insulin-induced inhibition of hepatic glucose production was blunted in HS-HS rats. The serum glucose level remaining for more time in high-levels at the postprandial period has important role on obesity-related hyperglycaemia.

There was a protective effect of NAC, normalizing the glucose response seen in the HS-HS compared with HS-N group (Figure 1). It has been shown that NAC prevented sucrose-induced insulin resistance [9, 10]. Researches in our laboratory have demonstrated that some antioxidants isolated from plants significantly reduced the blood glucose levels [2, 12]. However, in none of these studies, antioxidants were administered in obese rats due high-sucrose diet intake.

There is a growing awareness that variation in the ratio of starch to sucrose can affect the serum levels of lipids [12]. During periods of high-sucrose intake the hepatic tissue can convert glucose into fatty acids, from which triacylglycerols are made, transported to the blood steam as VLDL and stored as fat in adipose tissue [17]. Enhanced serum triacylglycerol, as observed in HS-HS rats (Table 3) is risk factor for myocardial infarction, arrhythmias and other cardiovascular complications [27]. It is widely accepted that HDL/TG ratio reflects information on LDL-composition, its size and density, since reduced HDL/TG ratio indicates VLDL and chylomicron remnants abnormally interaction with HDL, changing the clearance of these lipoproteins. Reduced HDL/TG ratio in HS-HS group (Table 3) indicated lower size of LDL-cholesterol, and small LDL-cholesterol is more susceptible to oxidative modification. Ox-LDL promotes atherosclerosis both by providing lipids signals that initially activate macrophages, and by stimulating foam cell formation [28].

NAC had beneficial effects on high-sucrose diet-induced dyslipidemia, enhancing HDL/TG ratio and reducing ox-LDL. Cholesterol levels enhanced in HS-HS group, and NAC reduced its level in the HS-N rats (Table 3). There is a growing awareness that total serum cholesterol level is a reflection of the individual lipoprotein level and metabolism [28]. HS-N had lower VLDL than HS-HS rats (Table 3) and VLDL is a LDL serum precursor [12]. Additionally, NAC secondary metabolites, as occurred with *S*-methylcysteine sulfoxide metabolites [29] could have properties depressing hepatic cholesterol synthesis. Moreover, NAC enhanced GSH-reductase activity (Table 3) thus shifting the NADPH, a co-factor for lipid synthesis, to GSSG reduction. Considering the importance of HDL to the reverse transport of cholesterol, and of ox-LDL on atherosclerosis, we can affirm that NAC improved the lipid profile in high-sucrose intake condition.

Judging from our experimental results, it is evident that the NAC effects on serum lipids, glucose tolerance and body weight were at least in part, antioxidant/oxidant mediated. First, the emerging view on the “antioxidant hypothesis” has indicated that ROS affect cellular signals [27]. ROS react with protein thiol moieties to produce a variety of sulfur oxidation states, thus diminishing the cellular uptake of lipids from the blood and changing lipid constituents of LDL, inducing LDL-oxidation. NAC reducing the oxidative stress and serum LH improved the lipid profile. Second, hyperglycemia, even within normal no diabetic range, is directly involved in pathogenic processes because it creates oxidative stress. NAC decreasing the oxidative stress, as showed by LH reduction and enhanced TAS in HS-N rats maintained the precise glucose-intake-expenditure, thus improving the glucose tolerance.

Several factors may be related to NAC effects on oxidative stress and decreased serum LH and ox-LDL levels. Both enzymatic and non-enzymatic antioxidant pathways may scavenge oxidative stress and so ox-LDL generation. During the reduction of hydrogen peroxide, GSH is oxidized to GSSG and the reduction of GSSG to GSH is catalyzed by GSH-reductase. The decreased serum GSSG in HS-N group compared to HS-HS (Table 3) indicated that NAC was used to inhibit LH production, thus reducing the GSH oxidation to GSSG, allowing increased GSH/GSSG ratio. NAC in HS-N rats enhanced GSH-reductase activity as compared to HS-HS, allowing the GSH reposition from GSSG reduction. Note that TAS was enhanced in HS-N rats. Cysteine is rate limiting for GSH synthesis and increased dietary cysteine prevented the high-sucrose induced decrease in hepatic GSH levels [15]. NAC is a thiol compound with high solubility [30], this fact can explain its action on lipid moiety of LDL, decreasing ox-LDL.

Curiously GSH-reductase activity was also enhanced in HS-HS group when compared to control rats. However, the maintenance of GSH, the enhanced GSSG and decreased GSH/GSSG ratio in HS-HS animals indicated that GSH was not enough to inhibit the ROS action on LDL. Therefore, ox-LDL and LH were enhanced in HS-HS rats. In HS-CN group, the ox-LDL, HDL and HDL/TG ratio normalization were associated with the synergistic effect of dietary changes and NAC intake. Note, Table 3, that TAS and GSH/GSSG were enhanced in these animals.

The significance of this study has been mainly explained by the potential application of its findings to human nutrition and preventive medicine. Since sucrose is one of the most important cornerstones of obesity incidence and its adverse effects, research is needed to answer an important question: What is the best dietary supplement to improve obesity-related effects disease?

In conclusion, NAC improved the high-sucrose diet-induced obesity and its effects on glucose tolerance, lipid profile, *in vivo* LDL-oxidation and serum oxidative stress, enhancing antioxidant defences. The application of this agent may be feasible and beneficial for high-sucrose diet-induced obesity, which certainly would bring new insights on obesity-related adverse effects control.

## Figures and Tables

**Table 1:** Effect of NAC on parameters related to obesity induced in male Wistar rats by high-sucrose diet.

Parameters	Groups	
	C Control (standard fed)	HS Obese (high-sucrose diet fed)
Initial body weight (g)	201.5 ± 5.2	202.0 ± 3.7
Final body weight (g)	295.1 ± 2.9	307.1 ± 2.8 ^a^
Body weight gain (g)	93.6 ± 1.2	125.1 ± 1.7 ^a^
Body mass index (g/cm^2^)	0.60 ± 0.03	0.66 ± 0.01 ^a^
Surface area (g^0.7^)	53.6 ± 1.1	57.6 ± 0.6 ^a^
Lee-index (g/cm)	0.30 ± 0.02	0.36 ± 0.01 ^a^

Values are given as mean ± SD of the mean.

^a^Values significantly different from C group, *P* < .001.

**Table 2:** General characteristics, nutritional parameters and glycemic response of the rats.

Determinations	Groups			
	C	HS-HS	HS-N	HS-CN
Initial body weight (g)	295.1 ± 7.5	301.5 ± 6.5	307.6 ± 6.3	304.6 ± 2.5
Final body weight (g)	388.7 ± 2.7 ^b^	446.4 ± 3.6 ^a,c,d^	403.7 ± 6.2 ^b^	407.5 ± 8.6 ^b^
Body weigh gain (g)	93.6 ± 3.5 ^b^	119.3 ± 3.2 ^a,c,d^	77.1 ± 14.3 ^b^	81.9 ± 11.6 ^b^
Food consumption (g/day)	28.04 ± 6.07 ^b,c,d^	21.29 ± 1.87 ^a^	18.76 ± 2.91 ^a^	21.11 ± 2.12 ^a^
Body mass index (g/cm^2^)	0.60 ± 0.02 ^b^	0.71 ± 0.01 ^a,c,d^	0.64 ± 0.03 ^b^	0.65 ± 0.03 ^b^
Surface area (g^0.7^)	64.9 ± 2.3 ^b^	71.6 ± 1.6 ^a,c,d^	66.7 ± 1.2 ^b^	67.2 ± 1.4 ^b^
Lee-index (g/cm)	0.29 ± 0.01	0.31 ± 0.01	0.29 ± 0.02	0.29 ± 0.01
Drinking solution ingestion (ml/day)	48.3 ± 7.9 ^b,c,d^	35.6 ± 8.6 ^a^	30.4 ± 2.4 ^a^	32.5 ± 2.1 ^a^
Energy intake (kcal/day)	80.2 ± 17.4 ^b,c,d^	68.8 ± 6.1 ^a^	60.6 ± 7.3 ^a^	60.4 ± 5.9 ^a^
Feed efficiency (%)	5.30 ± 0.52 ^b,d^	7.88 ± 0.61 ^a,c,d^	5.78 ± 0.48 ^b^	6.17 ± 0.43 ^a,b^
NAC intake (mg/day)	0.00 ^c,d^	0.00 ^c,d^	0.061 ± 0.001 ^a,b^	0.065 ± 0.002 ^a,b^
Glycemic response (mg/dl h)	70.5 ± 1.5 ^b^	87.1 ± 3.1 ^a,c,d^	63.5 ± 6.5 ^b^	60.4 ± 6.3 ^b^

C, control rats; HS-HS, rats receiving high-sucrose diet and water; HS-N, rats receiving high-sucrose diet and water for 22 days, giving NAC in its drinking water for more than 22 days; HS-CN, rats receiving high-sucrose diet and water for 22 days, giving NAC and control chow for more than 22 days.

Values are given as mean ± SD of the mean.

^a^Values significantly different from C group, *P* < .01;  ^b^Values significantly different from HS-HS group, *P*< .01;  ^c^Values significantly different from HS-N group, *P*< .01;  ^d^Values significantly different from HS-CN group, *P*< .01.

**Table 3:** Serum determinations.

Serum determinations	Groups			
	C	HS-HS	HS-N	HS-CN
Total protein (g/dl)	6.8 ± 0.2	6.7 ± 0.3	6.6 ± 0.1	6.1 ± 1.3
TG (mmol/l)	1.89 ± 0.17 ^b^	2.57 ± 0.19 ^a,c,d^	1.88 ± 0.23 ^b^	2.02 ± 0.21 ^b^
Cholesterol (mmol/l)	2.41 ± 0.02 ^c^	2.47 ± 0.01 ^c^	2.00 ± 0.02 ^a,b,d^	2.54 ± 0.09 ^c^
HDL (mmol/l)	1.04 ± 0.02 ^b,c^	0.68 ± 0.15 ^a^	0.71 ± 0.08 ^a^	1.01 ± 0.10 ^b,c^
VLDL (mmol/l)	0.87 ± 0.05 ^b^	1.18 ± 0.09 ^a,c,d^	0.86 ± 0.09 ^b^	0.93 ± 0.04 ^b^
LDL (mmol/l)	0.49 ± 0.04 ^b,c,d^	0.61 ± 0.11 ^a,c^	0.43 ± 0.02 ^a,b,d^	0.60 ± 0.10 ^a,c^
HDL/TG	0.24 ± 0.01 ^b,c^	0.11 ± 0.01 ^a,c,d^	0.17 ± 0.01 ^a,b,d^	0.22 ± 0.01 ^b,c^
ox-LDL (mg/dl)	35.1 ± 4.6 ^b,c,d^	56.4 ± 3.7 ^a,c,d^	45.8 ± 4.0 ^a,b^	42.1 ± 7.2 ^a,b^
LH (nmol/ml)	7.5 ± 0.2 ^b,c^	8.1 ± 0.1 ^a,c,d^	6.7 ± 0.2 ^a,b,d^	7.4 ± 0.1 ^b,c^
TAS (%)	62.1 ± 5.0 ^b,c,d^	44.8 ± 5.2 ^a,c,d^	79.2 ± 6.1 ^a,b^	78.9 ± 2.6 ^a,b^
GSH (nmol/ml)	15.0 ± 0.3	15.2 ± 0.1	15.3 ± 0.6	14.8 ± 02
GSSG (nmol/ml)	0.63 ± 0.02 ^c,d^	0.67 ± 0.02 ^c,d^	0.57 ± 0.01 ^a,b^	0.52 ± 0.02 ^a,b^
GSH/GSSG	23.8 ± 0.2 ^b,c,d^	22.7 ± 0.1 ^a,c,d^	26.8 ± 0.1 ^a,b,d^	28.5 ± 0.5 ^a,b,c^
GSH-reductase (nmol/ml)	0.25 ± 0.07 ^b,c^	0.36 ± 0.04 ^a,d^	0.41 ± 0.05 ^a,d^	0.22 ± 0.07 ^b,c^

C, control rats; HS-HS, rats receiving high-sucrose diet and water; HS-N, rats receiving high-sucrose diet and water for 22 days, giving NAC in its drinking water for more than 22 days; HS-CN, rats receiving high-sucrose diet and water for 22 days, giving NAC and control chow for more than 22 days.

Values are given as mean ± SD of the mean.

^a^Values significantly different from C group, *P* < .01;  ^b^Values significantly different from HS-HS group, *P*< .01;  ^c^Values significantly different from HS-N group, *P*< .01;  ^d^Values significantly different from HS-CN group, *P*< .01.
